# A journey through 80 years of Brazilian neurology

**DOI:** 10.1055/s-0043-1778007

**Published:** 2023-12-29

**Authors:** Ayrton Massaro, Hélio Teive

**Affiliations:** 1 Hospital Israelita Albert Einstein, São Paulo SP, Brazil.; 2 Universidade Federal do Paraná, Hospital de Clínicas, Departamento de Medicina Interna, Curitiba PR, Brazil.

*Arquivos de Neuro-Psiquiatria* (ANP) was first published in 1943, at a time when very few publications worldwide were dedicated to Neurology. This was made possible entirely due to the unwavering dedication of its founder, Dr. Oswaldo Lange. 1 As a result, the *Arquivos* has been the representative of Brazilian neurology, imparting it with a singular and authoritative voice. The efforts to promote Brazilian neurology on a global scale are evident in the years following, as demonstrated by the publication of distinguished international authors. 2 3 4 5 6

Under the leadership of Dr. Antonio Spina França Netto as the second Editor-in-Chief, ANP established itself as a platform for the Brazilian neurological community to report its groundbreaking scientific achievements in the subsequent years. 7 8 9 10

More recently, Drs. Luis dos Ramos Machado and José Antonio Livramento had the arduous task of amalgamating the ANP with the *Academia Brasileira de Neurologia* (Brazilian Academy of Neurology), making it the institution's main scientific publication. The *Academia Brasileira de Neurologia* 's main guidelines for the management and treatment of neurological diseases were successfully disseminated through this initiative. 4

Dr. Paulo Caramelli together with Dr Helio Teive have initiated a new cycle of challenges for the ANP by promoting greater interaction between national and international authors. This development necessitates a substantial transformation to accommodate the evolving concepts of scientific publishing within a global community that increasingly demands prompt access and dependability of information. 3 4

Following the guidelines set by Dr Lange, this special issue of the ANP brings together the contributions of distinguished Brazilian neurologists from various areas of neurological knowledge on topics of current relevance, with the accumulated experience of Brazilian neurology over the past 80 years.

Brazil's multi-ethnic population, characterized by significant socio-economic disparities, has posed significant challenges in the treatment of stroke patients. However, these difficulties did not impede the country's progress in the late 1990s when Brazil became the first nation in Latin America to introduce intravenous thrombolytic treatment for stroke patients. This move paved the way for the coordination and arrangement of care for these patients during the acute phase, enabling the disease to be addressed at various stages, from primary and secondary prevention to rehabilitation. Brazil has also played an active role in international stroke clinical trials since the early stages, contributing extensively to the field. Martins and colleagues 11 highlight the development of reperfusion treatment for acute ischemic stroke, taking into account Brazil's involvement in the planning of new clinical studies.

Kouyoumdjian and Estephan 12 embarked on an extensive examination of the fundamental principles that govern the neuromuscular junction, which is of paramount significance in comprehending current therapeutic approaches.

Post-COVID cognitive impairment is a condition characterized by cognitive dysfunction in multiple domains that occurs in individuals who have previously contracted severe acute respiratory syndrome coronavirus 2 (SARS-CoV-2) and cannot be attributed to any alternative diagnosis. It is crucial to identify the cognitive domains that are most affected to improve diagnosis and provide potential strategies for effective management. 13

Alzheimer's disease (AD) has undergone numerous revisions of its definition and diagnostic boundaries due to advances in our understanding of neurodegeneration and the development of sophisticated diagnostic techniques over the past several decades. In this issue of ANP Nitrini et al. reviewed the past of AD. 14

Barsottini and colleagues 15 have underscored the importance of clinical-neurological collaboration in the assessment and detection of neurological manifestations. Specifically, they utilized Sjogren's syndrome, a multisystem disorder rooted in autoimmune processes that impact the salivary and lacrimal glands and may affect the central and peripheral nervous systems, as a prime example. The higher prevalence in females and the tendency for symptoms that mimic other neurological conditions contribute to the diagnostic complexity of the syndrome.

The history and rationale of the development of new drugs for migraine treatment, an area of special interest for daily neurological practice, is expertly reviewed by Kowacs and colleagues. 16 Migraines pose a substantial burden on a large portion of the global population, causing significant disability. In addition to educational measures, treatment approaches have encompassed a range of acute and preventive medications for migraines. In countries like Brazil, it is essential to assess the cost-effectiveness of the medications employed, despite the various therapeutic options available.

On the other hand, Camargo et al. present a seminal article on the history of world neurology, with an emphasis on the father of modern neurology, the eminent professor Jean-Martin Charcot: the polymath. 17 Individuals who are classified as polymaths are those who have not confined themselves to a single area of knowledge and have instead pursued various opportunities by leveraging the advantages provided by technological advancements and the increasingly indistinct boundaries between scientific disciplines. Jean-Martin Charcot is undoubtedly one of those individuals who successfully bridged the diverse fields of knowledge during his era, laying the groundwork for contemporary neurology.

Neurovirology plays a crucial role in global health. Arboviruses, such as Dengue, Chikungunya, and Zika, pose a significant threat due to their mosquito-borne nature. Recently, a triple epidemic has occurred, causing neurological manifestations that require attention from the neurological community. In the area of neuro-infection, an extraordinary review of Dengue, Zika, and Chikungunya infections in the nervous system is presented by Puccioni-Sohler et al. 18

The application of precision medicine to neuroimmunology seeks to offer a highly precise and nuanced approach to management, by providing recommendations that are tailored to the specific disease subtype, clinical status, existing radiological and para-clinical data, and other biological markers. The field of neuroimmunology is continually advancing, with ongoing efforts to identify reliable biomarkers that can predict disease outcomes· Dos Passos and colleagues 19 present an article on the diagnosis and treatment of neuroimmunological diseases in the era of precision medicine.

Advances in the diagnosis of diffuse glial tumors of the central nervous system are presented by Godoy et al. 20 A very current topic, regarding the novelties of long-term epilepsy-associated tumors (LEATS), is presented by Rosemberg and colleagues. 21 In the area of Alzheimer's disease, Teixeira et al. 22 present a review of behavioral or neuropsychiatric symptoms, discussing psychopathology and management. A special article on the history of electroencephalography is presented by Caeira et al. 23 with an appraisal to Hans Berger by the time of his 150th birthday. Dach et al. 24 present a systematic review of the best evidence-based practice in low back pain, focusing on the treatment of myofascial pain with dry needling. Finally, Sobreira Neto and colleagues 25 discuss the diagnosis and treatment of REM sleep behavior disorder in a clear and up-to-date manner. Celebrating the 80th anniversary of the founding of Arquivos de Neuro-Psiquiatria, we wish all our colleagues in the field of neuroscience an excellent read.
